# The prevalence and predictors of domestic violence among pregnant women in Southeast Oromia, Ethiopia

**DOI:** 10.1186/s12978-019-0694-9

**Published:** 2019-03-25

**Authors:** Kalkidan Yohannes, Lulu Abebe, Teresa Kisi, Wubit Demeke, Solomon Yimer, Mohammed Feyiso, Getinet Ayano

**Affiliations:** 1 Department of Psychiatry, College of Medicine and Health Sciences, Dilla University, Dilla, Ethiopia; 2 School of Public Health, College of Medicine and Health Sciences, Nekemte University, Nekemte, Ethiopia; 3 Department of psychiatry, Amanuel Mental Specialized Hospital, Addis Ababa, Ethiopia

**Keywords:** Domestic violence, Pregnant women, Prevalence, Associated factor, Ethiopia, Häusliche Gewalt, Schwangere, Prävalenz, assoziierter Faktor, Äthiopien

## Abstract

**Introduction:**

Domestic violence is a common global health problem and relatively hidden and ignored form of violence against pregnant women. The magnitude of domestic violence among pregnant women is higher in low and middle-income countries including Ethiopia as compared with developed countries. Domestic violence is a violation of human right and associated with numerous adverse outcomes for mothers and the offspring. However, research on domestic violence and predictors against pregnant women is limited in Ethiopia. Therefore, the aim of this study was to assess the magnitude and predictors of domestic violence among pregnant women in southeast Oromia, Ethiopia.

**Methods:**

Cross-sectional study design was utilized among 299 pregnant women selected by systematic sampling technique. A structured World Health Organization (WHO) multi-country study questionnaire on women health and domestic violence was used to measure domestic violence. Binary and multivariable logistic regression models were fitted. Odds ratios (OR) with the corresponding 95% confidence interval (95%CI)) was computed to assess the strength of association.

**Result:**

The prevalence of domestic violence was 64.6% (CI: 58.5, 69.9%). Physical violence was reported as the commonest type of violence (44.1%) followed by psychological (39.1%) and sexual (23.7%) violence. In the multivariable analysis, being illiterate (OR = 6.3; 95%CI: 2.23, 11.65), Husband’s alcohol consumption (OR = 5.726; 95% CI 1.873, 11.51), husband history of arrest (OR = 2.59; 95% CI: 1.15, 5.88) and occupation of husband (farmer) (OR = 3.26; 95% CI: 1.29, 8.25) were significantly associated with domestic violence against pregnant women.

**Conclusion:**

This study revealed that a remarkable proportion of pregnant women had experienced domestic violence in their lifetime (64.6%). Being illiterate, husband’s alcohol consumption, occupation (farmer and self-employed), and history of arrest were significantly associated with domestic violence among pregnant women. The findings suggest screening for domestic violence among pregnant women visiting antenatal care clinic and early intervention based on the findings. Integrating health education program on domestic violence with the existing maternal health program is warranted.

## ABSTRAKT

**Einführung:**

Häusliche Gewalt ist ein allgemeines globales Gesundheitsproblem und eine relativ versteckte und ignorierte Form von Gewalt gegen Schwangere. Das Ausmaß häuslicher Gewalt unter schwangeren Frauen ist in Ländern mit niedrigem und mittlerem Einkommen einschließlich Äthiopien höher als in Industrieländern. Häusliche Gewalt ist eine Verletzung der Menschenrechte und ist mit zahlreichen nachteiligen Folgen für Mütter und Nachkommen verbunden. Die Forschung zu häuslicher Gewalt und Prädiktoren gegen schwangere Frauen ist in Äthiopien jedoch begrenzt. Ziel dieser Studie war es daher, das Ausmaß und die Prädiktoren für häusliche Gewalt unter Schwangeren im Südosten von Oromia, Äthiopien, zu bewerten.

**Methoden:**

Bei 299 schwangeren Frauen, die durch systematische Stichprobenverfahren ausgewählt wurden, wurde ein Querschnittsstudiendesign verwendet. Ein strukturierter, aus mehreren Ländern bestehender Fragebogen zur Weltgesundheitsorganisation (WHO) zur Gesundheit von Frauen und häuslicher Gewalt wurde zur Messung häuslicher Gewalt verwendet. Es wurden binäre und multivariable logistische Regressionsmodelle eingebaut. Zur Bewertung der Assoziationsstärke wurden Odds Ratios (OR) mit dem entsprechenden 95% -Konfidenzintervall (95% CI) berechnet.

**Ergebnis:**

Die Prävalenz häuslicher Gewalt lag bei 64,6% (CI: 58,5%, 69,9%). Körperliche Gewalt wurde als die häufigste Form von Gewalt gemeldet (44,1%), gefolgt von psychologischer (39,1%) und sexueller (23,7%) Gewalt. In der multivariablen Analyse, die Analphabeten ist (OR = 6,3; 95% CI: 2,23, 11,65), ist der Alkoholkonsum des Ehemanns (OR = 5726; 95% CI 1873, 11,51) die Anamnese des Anhaltens (OR = 2,59; 95% CI 1,15, 5,88) und der Beruf des Ehemanns (Landwirt) (OR = 3,26; 95% CI: 1,29, 8,25) waren signifikant mit häuslicher Gewalt gegen Schwangere verbunden.

**Fazit:**

Diese Studie ergab, dass ein bemerkenswerter Anteil schwangerer Frauen in ihrem Leben häusliche Gewalt erlebt hatte (64,6%). Als Analphabeten waren Alkoholkonsum, berufliche Tätigkeit (Landwirt und Selbständige) und Verhaftungsgeschichte des Ehemannes signifikant mit häuslicher Gewalt unter Schwangeren verbunden. Die Ergebnisse deuten auf ein Screening auf häusliche Gewalt bei Schwangeren in einer Klinik für vorgeburtliche Versorgung und eine frühzeitige Intervention basierend auf den Ergebnissen hin. Die Einbindung des Gesundheitserziehungsprogramms im Inland in das bestehende mütterliche Gesundheitsprogramm ist geboten.

## Plain English summary

Domestic violence (DV) in this study refers to physical, sexual, psychological or emotional violence or abusive behaviors that are directed to a pregnant woman by a family member (i.e. an intimate male partner, marital/cohabiting partner, parents, siblings, or a person very well known to a family or significant other such as former partner) when such violence often takes place in home. DV is a global public health problem and is linked with physical, psychological and economic consequences. DV is also associated with series adverse health outcome for the affected women as well as their offspring. However, there are limited studies which assessed the magnitude of DV against pregnant women in Ethiopia. Therefore, this study was conducted to determine the prevalence and associated factors DV during pregnancy southeast Oromia, Ethiopia.

A systematic random sampling technique was used to select the study participants. The participants were asked about their sociodemographic, clinical and substance-related factors. Additionally, we measured the DV by asking the participants the structured questionnaire which was used in previous studies in Ethiopia. The study included two hundred ninety -nine (299) participants which were selected randomly.

The results of the current study revealed that nearly two-thirds (64.6%) of pregnant women had experienced DV in southeast Oromia, Ethiopia. The most commonly experienced form of DV was physical violence (44.1%) followed by psychological (39.1%) and sexual (23.7%) violence.

Regarding the associated factors, those pregnant women who were illiterate, whose husband had a history of alcohol consumption, work as a farmer and reported a history of arrest for a husband were more likely to experience DV. Screening for domestic violence among pregnant women visiting antenatal care clinic and appropriate intervention, and it’s also better to integrate health education program on domestic with a maternal health program.

## Background

Domestic violence is a form of violence against women which the United Nations Declaration on the Elimination of Violence Against Women defines as “any act of gender-based violence that results in, or is likely to result in, physical, sexual or psychological harm or suffering to women, including threats of such acts, coercion or arbitrary deprivation of liberty, whether occurring in public or in private life” [1]. Violence against women by partners is referred to as domestic violence (DV), spousal assault, intimate partner violence (IPV), wife abuse, wife assault, and battered wife syndrome [2].

Domestic violence among women is a global issue and [3], a significant public health problem, as well as a fundamental violation of women’s human rights [4], crossing cultural, geographic, religious, social and economic boundaries [5]. Domestic violence is the most prevalent yet relatively hidden and ignored form of violence against women and girls [6]. One in every four women will experience domestic violence in her lifetime [7]. The prevalence of domestic violence during pregnancy varies and has been found in 3–13% of pregnancies in many studies around the world [8], while in developing countries ranges from 4 to 29% [3].

In 2013 a more recent analysis based on existing data from over 80 countries, found that globally 35% of women have experienced either physical and/or sexual intimate partner violence or non-partner sexual violence. Most of this violence is intimate partner violence [4]. According to this study, globally, 38% of all murders of women are committed by intimate partners [4]. Domestic violence against women leads to far-reaching physical and psychological consequences, some with fatal outcomes [6].

Domestic violence has been found to be associated with hyperemesis, head and back pain during pregnancy and poor obstetric outcome [9]. Women who were beaten during pregnancy by their partners are 48% more likely to be infected with HIV/AIDS. Violence against women by partners during pregnancy is a major public health concern [10]. It causes serious health consequences for both mother and child. It leads to high-risk pregnancies and pregnancy-related problems, including miscarriage, pre-term labor and low birth weight [11]. Currently available data indicate that pregnant women who are experienced domestic violence are at increased risk for developing certain mental health problems [12] and the impact of domestic abuse in pregnancy can be long-lasting physical disability and/or psychological (including depression, anxiety, post-traumatic stress disorder, flashbacks, nightmares or an exaggerated startle response) [13].

According to the analysis of prevalence data from 19 countries in 2010 on Intimate partner violence during pregnancy, the prevalence of intimate partner violence during pregnancy among ever-pregnant women ranged from approximately 2% in Australia, Denmark, Cambodia, and the Philippines to 13.5% among ever-pregnant women in Uganda [14].

Financial problems (38.3%), interpersonal conflicts (19.8%), and alcohol use (10.7%) were reported as the most important factors leading to violence [15–19]. In a study conducted in Turkey, 53.8% of the participants reported violence and educational deprivation will be solved by allowing women to work in a paid job and advance professions [20].

In a study conducted among pregnant women in Turkey, regular smoking, unwanted pregnancy, low education level of husband, low family income and being in the second trimester were determined to be the main predictors of overall violence during pregnancy [20].

Furthermore, in a study conducted in Brazil including 1379 pregnant women found that adolescent intimate partner and witnessing physical aggression before 15 years of age were found to be linked with psychological violence. Whereas, having an intimate partner who uses drugs and does not work were linked with physical/sexual violence. Moreover, low level of education, having a history of physical aggression during childhood and intimate partner consumes alcoholic beverage twice or more weekly were linked with psychological and physical/ sexual violence [19].

Factors that were found to be strongly associated with domestic violence on the part of the husbands were low educational level, unskilled workers, alcohol consumption and multiple sexual partners. On the part of the women, the factors were alcohol consumption, and being HIV positive [21–24].

According to Community based studies conducted among childbearing age group in different parts of Ethiopia indicated; the prevalence of different forms of domestic violence experienced by women 50.8% around Gondar in northwest Ethiopia and 78% in Awi zone Fagitale Koma woreda. The Prevalence of physical violence was found to be 32.2%, while that of forced sex and physical intimidation amounted to 19.2 and 35.7%, respectively around Gondar in northwest Ethiopia and about 73.3, 58.4 and 49.1% of women reported different forms of psychological, physical and sexual violence, respectively in Fagitalekoma Woreda, Awi zone [25, 26].

Study conducted in Butajira between August to October 2001 on Women’s Health and Life Events in Rural Ethiopia involved 3200 women among them 214 (10%) of ever-married women were pregnant at the time of the interview, and of these pregnant women, 164 (77%) reported physical violence by a partner during the current pregnancy. Among women who had been abused during pregnancy, forty-six (28%) had been punched or kicked on the abdomen. In almost all cases (98%), the perpetrator of the abuse was the father of the child, as a whole factor associated with domestic violence among pregnant women were not studied [27].

Similar community-based studies done in some parts of Oromia regional state showed that prevalence of domestic violence against women; 39.7% in Kersa woreda eastern Oromia [28] and 76% around Nekemte western Oromia. On the other hand, 68.6%of women experienced at least one or more incidents of physical violence in their lifetime, and the prevalence of psychological violence was 70.2%, during lifetime and 63.9%, in current experiences and 62.6% the incidents have happened during the past 12 months. About 59% of the respondents reported that at some point in their lifetime, their husbands/partners had forced them to have sexual intercourse without their interest or consent and in 51% of them it has happened in the preceding 12 months [29].

Despite this significant health problem, previous studies conducted in Ethiopia focused on the magnitude and associated factors of DV against women in general. Hence, studies focused on the magnitude of domestic violence and predictors is against pregnant women are limited in Ethiopia as well as in the study area. Therefore, the aim of this study was to assess the magnitude and predictors of domestic violence among pregnant women in southeast Oromia, Ethiopia.

## Methods

### Study design, period, setting, and population

An institutional-based cross-sectional study was conducted from April to May 2014 in Gedo hospital, which is found in Oromia regional state of West Shewa zone, Ethiopia. It is located in central Ethiopia at a distance of 176-km away from the capital city, Addis Ababa. Gedo hospital is one of the newly established hospitals in 2010 by the collaboration of the Ethiopian Ministry of health and Oromia Regional Health Bureau to serves the catchment area population. As the document in the hospital shows, monthly an average of 800 pregnant women attends ANC clinic at the hospital.

The study population consisted of all pregnant women attending the antenatal care unit Gedo hospital during the data collection period. Those pregnant women who were critically ill were excluded from the study.

### Sample size and sampling technique

Sample size (n) was calculated based on single population proportion formula, by assuming 95% confidence level, the prevalence of domestic violence among pregnant women which was found to be 77% in Butajira rural health study and a precision of 5% between the sample and the parameter was taken. α =0.05(95%) =1.96

$$ \frac{n={\left( z\alpha /2\right)}^2\mathrm{x}\ \mathrm{p}\left(1-\mathrm{p}\right)}{{\mathrm{d}}^2} $$

$$ \frac{n={(1.96)}^2\mathrm{x}\ 0.77\left(1-0.77\right)}{0.05^2}=272 $$

By considering a 10% non-response rate the final sample size was 299.

We used a systematic sampling technique to select the two hundred and ninety-nine (299) pregnant women who were included in our survey. We determined the sampling interval by dividing the total study population who had to follow up during the 1-month data collection period (800) by total sample size (299). Therefore, the sampling fraction is 800/299 ≈ 3. Hence, the sample interval is 3. We selected the first study participant by lottery method and the next study participants were chosen at regular intervals (every 3rd interval) and interviewed by data collectors.

### Data collection tools and procedures

Data were collected by trained nurses by face-to-face interviewing of the participant (women who were currently pregnant). The questionnaire was pre-tested by taking 5% of the calculated sample size. The questionnaire contained socio-demographic characteristics (age, income, education, occupation, marital status, and others). semi-structured questionnaires were used to collect data on clinical factors.

Data on the magnitude of domestic violence was collected through interview using standard and structured WHO multi-country study questionnaire on women health and domestic violence questionnaire. Domestic violence was recoded into a dichotomous variable to denote the presence and absence of domestic violence in their lifetime. The WHO violence against women instrument and version for this study contains 6 questions for physical abuse, 3 questions for psychological abuse and 3 questions for sexual abuse.

To assess the experience of physical abuse, pregnant women were asked whether they were ever slapped, hit or beaten in the previous and index pregnancy, ever punched or kicked in the abdomen while pregnant, hit with a fist or with something else that could hurt her, Chocked or burnt on purpose and threatened with gun, knife or another weapon.

Then physical violence was computed by aggregating these 6 items using SPSS 20.00 transformation functions. At least one YES response among the six items qualifies the respondent for being faced with any form of physical violence [30]. Three items were inquired about psychological violence practices against women; being insulted by their husband/partner using abusive language; ignored or shown indifference by their husband/partner and experienced suspicion or accused her as she was unfaithful by the husband. All women who experienced the item in question were asked the perceived violence in the past 12 months. Then psychological violence was computed by aggregating these three items using SPSS 20.00 transformation functions. At least one YES response among the three items qualifies the respondent for being faced with any form of psychological violence [30].

Respondents who answered “Yes” to at least one of the listed questions in relation to the previous or current pregnancy were considered to have experienced domestic violence and those answering “No” to all of the questions were considered as not having experienced violence. Those who had experienced domestic violence were then asked about the perpetrator(s).

In this study, substance use such as alcohol indicates lifetime use of those substances. Those participants who had a history of substance use in their lifetime were considered as positive for substance use.

### Data quality control issues

Training was given to the data collectors and supervisors on the data collection tool and sampling techniques by the researcher. Supervision was held regularly during the data collection period both by the researcher, co-investigators and supervisors to check on a daily basis for completeness and consistency.

### Data analysis

Data were analyzed using SPSS version 20. Description statistics (frequencies, proportions, means, and standard deviations) were used to present the sociodemographic and the prevalence of domestic violence. Both bivariate and multivariate logistic regression analysis were carried out to see the association of each independent variable with the outcome variable. A *p*-value of less than 0.05 was considered statistically significant, and an adjusted odds ratio with 95% CI was calculated to determine the association.

### Ethical consideration

We obtained ethical clearance after approval from the Institutional Review Board (IRB) of the College of Medicine and Health Sciences, the University of Gondar and from Amanuel Mental Specialized Hospital. The data collectors have clearly explained the aims of the study to the study participants. Information was collected after obtaining written consent from each participant. Written informed consent was obtained from the informants and the comparison respondents/subjects after they were received oral information about the study, including an assurance that they could withdraw from the study at any time. Confidentiality was maintained through the study. Participants who were found to have DV were referred for further investigations and support.

## Result

A total of 299 participants were included in this study. The mean age of women was 27.72 SD (±6.32) years. Majority of the study participants were Oromo in ethnicity (93%) and nearly two-thirds (62.9%) of women were protestants. Around half (47.5%) of women were illiterate and 218(72.9%) were housewives (Table 1).

**Table 1:** Socio-demographic characteristics of pregnant women attending antenatal care clinic at Gedo hospital west shewa, Oromia, Ethiopia, March 2014 (*n* = 299)

variable		frequency	Percent
Age of women	18–27	163	54.5
	28–37	113	37.8
	38–50	23	7.7
Religion	Protestant	188	62.9
	Orthodox	92	30.8
	Others	19	6.4
Marital status of women	married	280	93.6
	Single or separated	19	6.4
Educational status of women	Illiterate/no education	142	47.5
	primary school education	85	28.4
	secondary school	44	14.7
	college diploma & above	28	9.4
Occupation of women	housewife	218	72.9
	trading/merchant	34	11.4
	employee	26	8.7
	day laborer	21	7.0

### Domestic violence against pregnant women

Among the total pregnant women included in the study, nearly two-thirds 193(64.6%) of them reported that they had ever experienced at least one type of domestic violence during their previous pregnancies, while they were pregnant. Among these 132(44.1%), 117(39.1%) and 71(23.7) women were experienced physical, psychological or sexual violence respectively. One hundred two (34.1%) were experienced domestic violence during the index pregnancy. Twenty-five (8.4%) women reported psychological, physical and sexual violence attempted and / committed at the same time *by* their husband (figare2). The most common offender was the Husband/partner of the women (88%).

### Domestic physical violence

One hundred thirty-two (44.1%) women reported that they had experienced different forms of physical violence while they were pregnant, and women’s husbands/partners (96.5%) were the major offender for the domestic violence. Among these women: 107(35.8%) reported ever slapped,hit or beaten while pregnant,62(20.7%) reported being slapped, hit or beaten in the last /in index pregnancy,8(2.7%) reported ever punched or kicked in the abdomen while pregnant, 44(14.7%) reported being hit with a fist or with something else that could hurt them, 10(3.3%) reported being threatened with gun, knife or other weapon and 3 (1%) reported being chocked or burnt on purpose (Table2).

### Domestic psychological violence

One hundred seventeen (39.1%) women reported different forms of psychological violence against them. Among these insulting by using abusive language (35.1%), ignored h or shown indifference (22.7%), expressed suspicion or accused there as they were unfaithful (19%) were reported by women respectively (Table 2).

**Table 2:** Distribution of physical, psychological and sexual violence among pregnant women Gedo, Ethiopia, 2014 (*n* = 299)

Domestic violence	Frequency	Percent
Physical violence		
Ever slapped, hit or beaten while pregnant/previous pregnancy	107	35.8
Slapped, hit or beaten in the index pregnancy	62	20.7
Ever punched or kicked in the abdomen while pregnant	8	2.7
Hit with a fist or with something else that could hurt her	44	14.7
Chocked or burnt on purpose	3	1
Threaten with a gun, knife or another weapon	10	3.3
Psychological violence		
*Insulted by using abusive language*	105	35.1
*Ignored or shown indifference by her husband/partner*	68	22.7
*Experienced suspicion or accused her she was unfaithful*	58	19.4
Sexual violence		
Physically forced her to have sexual intercourse against her will	64	21.4
Ever have sexual intercourse she didn’t want because she was afraid	24	8.0
Anyone ever touched her sexually in a way she didn’t approve	40	13.4
Physical, psychological & sexual violence overlapped	25	8.4
Domestic violence during index pregnancy	107	34.4

### Domestic sexual violence

Seventy-one (23.7%) women reported that they had experienced different forms of sexual violence by their husband while they were pregnant. Among these women (21.4%) physically forced to have sex when they didn’t want to have sexual intercourse, (8%) ever have sexual intercourse that they didn’t want because they were afraid and (13.4%) women ever have touched their sexuality in a way they didn’t approve (Table2).

### Factors associated with Domestic violence of pregnant women

Multivariable logistic regression revealed being illiterate, husband’s alcohol consumption, occupation (farmer), and history of arrest were significantly associated with domestic violence among pregnant women. Illiterate mothers were about 6 times more likely to be violated than mothers whose education levels were diploma and above [AOR =6.27; 95%CI: 2.23, 11.65]. On the other hand, mothers whose education levels primary school were 4.73 times more likely to be experienced domestic violence compared to those pregnant women whose education were diploma and above [OR = 4.73; 95%CI: 1.69, 13.20]. Similarly, domestic violence was 3 times [OR = 3.65; 95 CI: 2.01, 6.63] more likely to occur among those participants who have witnessed their maternal violence as a child.

The odds of domestic violence among those pregnant women having a husband who consumes alcohol was 5.72 times [OR = 5.726; 95% CI 1.873, 17.507] higher than those whose women husband did not use the substance. The odds of experiencing domestic violence among women who had a husband working as a farmer was 3.26 times higher than those pregnant women whose husband was government employee [OR = 3.26;95%CI:1.29,8.25]. In addition, those husbands working as day laborer and self-employed were 4 times[OR = 4.3;95% CI:1.69,10.79] more likely violated against their wife than government employee and husband/partner those who had have parental history of violence as a child 6 times (OR = 6.21;95%CI: 3.26,11.82) more likely violated against their women than those who have no history of parental violence as a child. Finally, partners who had a history of arrest was 2 times (OR = 2.59; 95 CI: 1.15, 5.88) more likely to be violated against their wife than those who have no arrested history (Table 3).

**Table 3:** Factors associated with domestic Violence among pregnant women, Gedo, Ethiopia, 2014 (*n* = 299)

Variables		Domestic Violence		COR with 95% CI	AOR with 95%CI
		Yes No			
Women education	Illiterate	98	40	5.39(2.34,12.40)	6.27 (2.23,11.65) *
	Primary school	55	30	4.03 (1.69,9.63)	4.73 (1.69,8.18) *
	Secondary school	30	14	3.86 (1.42,10.48)	2.3 (2.27,12.42) *
	diploma & above	10	22	1.00	1
Women occupation	House wife	143	75	2.60 (1.14,5.94)	1.98 (0.89, 4.78)
	Merchant &self-employ	39	16	3.32 (1.26,8.78)	2.43 (0.92, 7.20)
	Government employ.	11	15	1.00	1
Partner education	Illiterate	92	47	3.92 (1.75,8.75)	2.86 (0.98, 8.34)
	Primary school	53	22	4.82 (2.0,11.59)	3.27 (0.97, 10.97)
	Secondary school	36	15	4.80 (1.87,12.31)	3.18 (0.95. 11.78)
	diploma & above	11	22	1.00	
Partner occupation	Farmer	113	55	4.45 (2.09,9.48)	3.26 (1.29,8.25) *
	Merchant &self-employee.	68	25	5.9 (2.59,13.43)	4.3 (1.7,10.80) *
	Government employ.	12	26	1.00	1
Women Alcohol use	No	125	92	1.00	1
	Yes	68	14	3.57 (1.89,6.75)	2.24 (1.05,4.80) *
Husband- alcohol use	No	149	100	1.00	1
	Yes	44	6	4.92 (2.02,11.98)	5.73 (1.87,11.51) *
Women family history of violence	No	30	51	1.00	
	Yes	163	55	5.04 (2.92,8.68)	3.65 (2.01,6.63) *
Partner family history of violence	No	83	90	1.00	1
	Yes	110	16	7.45 (4.08,13.63)	6.21 (3.3,11.8) *
Partner arrested history	Yes	41	5	2.60 (1.24,5.41)	2.59 (1.15,5.88) *
	no	152	101	1.00	1

Key: * = p < 0.05 (significant association), ref [1] *reference category, COR crude odds ratio, AOR adjusted odds ratio*

## Discussion

In this study, the prevalence of domestic violence among pregnant women and their possible association with various variables were assessed. The results from the current survey revealed that a remarkable proportion of pregnant women had experienced domestic violence in their lifetime. Nearly two-thirds of pregnant women (64.6%) experienced domestic violence during their previous pregnancies. The magnitude of this study is in line with the study conducted in Tehran o (59%) [16], Bahrain (59%) [21], and Ethiopia (71%) [15].

Contrarily, the magnitude of domestic violence in this study was quite higher than the previous study results in Ethiopia which reported the prevalence between 39.7 to 58% [26–29]. Furthermore, the prevalence of domestic violence in the current study was lower than the findings from 80 countries analysis of as evaluated by WHO (34.1%) [4], South Africa (31%) [17], Kenya 37% [23], and in Nigeria (36%) [18]. The possible reason the observed variation might be the difference in educational level, cultural variations, and the accessibility of information on gender-related issues, reproductive health, violence as well as human rights.

Regarding the specific types of violence, the physical violence among pregnant is quite higher 2 to 10% more when compared with a study done in different countries in the world such as the Global 2010 analysis, South Africa, Brazil, Iran, and Gondar in Ethiopia [14, 16, 17, 19, 25] and quite much less when compared to study founding’s of eastern city of Turkey, Butajira,Ethiopia [20, 29] . The differences may be due to variations in cultural norms, and the other possible reason might be the definition of domestic violence by a different study conducted and the tool used to collect data could not be the same.

Concerning about the prevalence of different forms of psychological violence experienced by pregnant women (39.1%), in this study is in line with a study reports around Gondar zuria district, and South Africa Kwazulu, revealed that three eightieths to half of the women (35.7 to 49.5%) had experienced physical violence while they were pregnant [17, 27]. Nevertheless, it is quite lower than when it compared with a study done in Fagitale Koma woreda, Awi zone and Nekemte western Oromia [28, 30]. The possible reason for the difference might be study design, sample size, and the current study conducted at an institution among pregnant women attending ANC that might be discomforting to disclose violence outside their home.

Regarding sexual violence, the most frequent type of sexual violence reported by women was physically forced to have sex when they didn’t want to have sexual intercourse. This is incomparably higher than findings from a study conducted in the eastern city of Turkey and the Brazilian institutional study [19, 20]. Nevertheless; it is much lower than a study done in Fagitale Koma woreda Awi zone, around Nekemte western Oromia and north-central Nigeria [26, 28, 30], and in line with a study conducted around Gondar, northwest Ethiopia, that shows one in five women were experienced sexual violence by their partners [27]. The observed difference might be due to traditional gender norm and cultural differences that might do not support to disclose sexual violence. The physical violence among pregnant is quite higher 2 to 10% more when compared with a study done in different countries in the world such as the Global 2010 analysis, Kwazulu Natal South Africa, Brazil, Iran, around Gondar district [14, 16, 17, 19, 25];and quite much less when compared to study founding’s of eastern city of Turkey, Butajira,Ethiopia [20, 29] . The differences may be due to variations in cultural norms, study design, sample size, and the other possible reason might be the definition of domestic violence by a different study conducted and tool used to collect data could not be the same. Concerning about the prevalence of different forms of psychological violence experienced by pregnant women (39.1%), in this study is in line with a study reports around Gondar zuria district, and South Africa Kwazulu, revealed that three eightieths to half of the women (35.7 to 49.5%) had experienced physical violence while they were pregnant [17, 27]. Nevertheless, it is quite lower than when it compared with a study done in Fagitale Koma woreda, Awi zone and Nekemte western Oromia [28, 30]. The possible reason for the difference might be the current study conducted at an institution among pregnant women attending ANC that might be discomforting to disclose violence outside their home. Regarding sexual violence, the most frequent type of sexual violence reported by women was physically forced to have sex when they didn’t want to have sexual intercourse. This is incomparably higher than findings from a study conducted in the eastern city of Turkey and the Brazilian institutional study [19, 20]. Nevertheless; it is much lower than a study done in Fagitale Koma woreda Awi zone, around Nekemte western Oromia and north-central Nigeria [26, 28, 30], and in line with a study conducted around Gondar, northwest Ethiopia, that shows one in five women were experienced sexual violence by their partners [27]. The observed difference might be due to traditional gender norm and cultural differences that might do not support to disclose sexual violence.

In this study, the husband’s alcohol consumption was significantly associated with domestic violence among pregnant women. Alcohol consumers were more violated against their wife than non-consumers, and this could be explained by the fact that alcohol has an effect on neurotransmitters. This finding is similar to other study findings such as Brazil, Tehran and Turkey [16, 19, 20].

The current study also found that the husband’s occupational status was significantly associated with domestic violence. Farmers and self-employed were more likely violated against their women than a government employee. Probably, the stress of job and stress of business and trade in the private sector increase the risk of abusing the wives by their husbands. Other studies on the associated factors of domestic violence showed the same result [16, 24].

Moreover, pregnant women and their husband/partner have had witnessed their maternal violence as a child were associated with domestic violence against women. This could be due to the reason that parental behaviors were learned by children’s and might be practiced in their adult life. And this finding is in line with other study findings [19, 25]. Also, women’s husbands/partners previous history of arrest was strongly associated with domestic violence against women. This result has been reported in another study too [16, 25]. The precise reason for this might be most men with a history of arrested have other risk factors for violating against women, such as a low level of education, aggressive behavior, and so on.

Finally, the low level of women’s education was highly associated with domestic violence. This can be explained by the fact that educated women have the knowledge about their rights and relationships and can control their behaviors properly; and this study is in line with other studies [16, 17, 19, 20].

### The implication for future research and clinical practice

This study has found some implications for future research and clinical practice. First, in our study a remarkable proportion of women had experienced DV during pregnancy which implies the necessity of screening of DV among women in the ANC setting and the possible early interventions based on the findings; Second, the integration of educations DV into the existing maternal health program is recommended; thirdly, future longitudinal studies focusing on the basic determinants of DV among pregnant women are warranted.

### The strength and limitation of the study

This study has several strengths. First, the sample is adequate and from a well-defined catchment area. Second, we used the standardized instrument for measuring PTSD (standard and structured WHO multi-country study questionnaire on women health and domestic violence).

The limitation of this study includes not measuring the effects of the existing mental disorders which may overestimate the odds of exposure in our results. These could have an influence on these results. Another limitation is the association between different factors and DV does not imply causation due to the cross-sectional nature of the study. As this study is a cross-sectional study the occurrence of recall bias needs to be considered which might affect the observed prevalence.

## Conclusion

In summary, this study revealed that a remarkable proportion of pregnant women had experienced domestic violence in their lifetime (64.6%). Being illiterate, Husband’s alcohol consumption, occupation (farmer and self-employed), and history of arrest were significantly associated with domestic violence among pregnant women. The findings suggest screening for domestic violence among pregnant women visiting antenatal care clinic and appropriate intervention, and it’s also better to integrate health education program on domestic violence with a maternal health program.
